# Gallic Acid Ameliorates Angiotensin II-Induced Atrial Fibrillation by Inhibiting Immunoproteasome- Mediated PTEN Degradation in Mice

**DOI:** 10.3389/fcell.2020.594683

**Published:** 2020-10-30

**Authors:** Dan Han, Qi-Yu Zhang, Yun-Long Zhang, Xiao Han, Shu-Bin Guo, Fei Teng, Xiao Yan, Hui-Hua Li

**Affiliations:** ^1^Department of Cardiology, Institute of Cardiovascular Diseases, First Affiliated Hospital of Dalian Medical University, Dalian, China; ^2^Emergency Medicine Clinical Research Center, Beijing Chao-Yang Hospital, Beijing Key Laboratory of Cardiopulmonary Cerebral Resuscitation, Capital Medical University, Beijing, China

**Keywords:** gallic acid, atrial fibrillation, atrial structural remodeling, immunoproteasome, PTEN/AKT1

## Abstract

Atrial fibrillation (AF) is the most prevalent cardiac arrhythmia and is a major cause of stroke and heart failure. We and others have found that gallic acid (GA) plays a beneficial role in cardiac hypertrophic remodeling and hypertension. However, the effect of GA on angiotensin II (Ang II)-induced AF and atrial remodeling as well as the underlying mechanisms remain unknown. AF was induced in mice by Ang II infusion (2000 ng/kg/min) for 3 weeks. Blood pressure was measured using the tail-cuff method. Atrial volume was evaluated by echocardiography. Atrial remodeling was studied using hematoxylin and eosin, Masson’s trichrome, and immunohistochemical staining. Atrial oxidative stress was assessed by dihydroethidium staining. The gene expression of fibrotic and inflammatory markers and protein levels of signaling mediators were measured by quantitative real-time PCR and western blot analysis. In mice, GA administration significantly attenuated Ang II-induced elevation of blood pressure, AF incidence and duration, atrial dilation, fibrosis, inflammation, and oxidative stress compared with the vehicle control. Furthermore, GA downregulated Ang II-induced activity and expression of immunoproteasome subunits (β2i and β5i), which reduced PTEN degradation and led to the inactivation of AKT1 and downstream signaling mediators. Importantly, blocking PTEN activity by VO-Ohpic markedly reversed the GA-mediated protective effects on Ang II-induced AF and atrial remodeling. Therefore, our results provide novel evidence that GA exerts a cardioprotective role by inhibiting immunoproteasome activity, which attenuates PTEN degradation and activation of downstream signaling, and may represent a promising candidate for treating hypertensive AF.

## Introduction

Atrial fibrillation (AF) is the most prevalent cardiac arrhythmia and is associated with an increased risk of stroke, heart failure, and all-cause mortality (Staerk et al., 2017). AF is typically preceded by conduction abnormalities and structural remodeling, which is characterized by increased atrial fibrosis and dilation (Andrade et al., 2014). The renin–angiotensin system (RAS) plays an important role in the development of AF. As the main effector of the RAS, Ang II activates AKT1-mTOR, TGF-β1-Smad2/3, NF-κB, and NADPH oxidase through Ang II type 1 receptor (AT1R), which in turn promotes fibrosis, inflammation, production of reactive oxygen species, and an abnormal ion channel function in the atrium, thereby leading to the occurrence of AF (Gao and Dudley, 2009; Hu et al., 2015). Therefore, AT1R is the central mediator of atrial remodeling and AF, and thus represents a key target for early treatment of AF. Recent studies have demonstrated that phosphatase PTEN (a phosphatase and TENsin homolog deleted from chromosome 10) exhibits negative regulation of AT1R-induced signaling pathways and AF (Li et al., 2018, 2019). Importantly, we and others have revealed that the activity of PTEN is restored by natural compounds such as resveratrol and indole-3-carbinol (Chen et al., 2019; Lee et al., 2019; Zou et al., 2019). Thus, regulation of PTEN represents a promising strategy for treating cardiovascular disease.

Gallic acid (GA) is a natural phenolic acid that is abundant in several plants. Accumulating evidence has demonstrated that GA plays a beneficial role in apoptosis, hypertension, cardiac hypertrophy, and fibrosis (Ryu et al., 2016; Jin et al., 2017, 2018a,b). GA improved isoproterenol-induced cardiomyocyte hypertrophy and fibrosis by inhibiting the JNK2-Smad3 signaling pathway (Ryu et al., 2016). Moreover, GA also reduced cardiac oxidative stress and hypertension by regulating GATA4-NOX signaling in spontaneously hypertensive rats (Jin et al., 2017). Recently, we found that administration of GA attenuated pressure overload-induced cardiac hypertrophic remodeling and heart failure, which are associated with the autophagy-dependent degradation of hypertrophic mediators (EGFR, gp130, and calcineurin A) and the suppression of downstream signaling cascades (Yan et al., 2019). Moreover, GA ameliorated hypertension and vascular remodeling in Ang II-treated mice by suppressing the immunoproteasome-dependent degradation of endothelial nitric oxide synthase (Yan et al., 2020). However, the cardioprotective effect of GA against Ang II-induced AF and the underlying mechanism remain unknown.

In this study, we revealed that GA significantly reduced AF incidence and duration as well as atrial structural remodeling in Ang II-treated mice. Mechanistically, the protective effect is associated with suppression of the activity and expression of the immunoproteasome subunits β2i and β5i, which inhibit PTEN degradation and AKT1 activation, resulting in the downregulation of the TGF-β1-Smad2/3 and NF-κB signaling pathways. Collectively, our results suggest that GA is a novel regulator of the immunoproteasome and PTEN stabilization, and may provide a potential treatment option for hypertensive AF.

## Materials and Methods

### Animal Models and Experimental Protocols

C57BL/6 mice (male, 8–10 weeks of age, *n* = 200) were used to establish the model of AF by subcutaneous infusion of Ang II (2000 ng/kg/min; Sigma-Aldrich, St. Louis, MO, United States) or saline for 3 weeks as described previously (Li et al., 2018, 2019; Zhang et al., 2020). The tail-cuff system (Softron BP98A; Softron Tokyo, Japan) was used to assess the systolic blood pressure (SBP) as previously described (Wang et al., 2016, 2018; Zhang et al., 2020). All animal studies were approved by the Animal Care and Use Committee of Dalian Medical University (No. LCKY2016-31) and conformed to the Guide for the Care and Use of Laboratory Animals published by the United States National Institutes of Health (NIH Publication No. 85-23, revised 1996).

### Dosage Information

Animals were orally gavaged with vehicle or GA (5 or 20 mg/kg BW/day, Sigma-Aldrich, United Kingdom) 1 day before Ang II infusion. The specific PTEN inhibitor VO-OHpic (Selleck, Houston, TX, United States) was intraperitoneally administered (10 mg/kg/day per mouse) in mice beginning 1 day before Ang II infusion (Chen et al., 2019).

### AF Induction

Mice were anesthetized with 2.5% tribromoethanol (0.02 mL/g; Sigma-Aldrich, United Kingdom) by intraperitoneal injection. A Millar 1.1 F octapolar EP catheter (Scisense, NY, United States) was guided into the right atrium and ventricle. AF Inducibility was gauged by applying a 5-s burst using the automated stimulator as described previously (Li et al., 2018, 2019; Zhang et al., 2020). The 1-lead body surface ECG and ≤4 intracardiac bipolar electrograms were recorded by the 15 A computer-based data acquisition system (GY6328B; HeNan HuaNan Medical Science and Technology, Co., Ltd.). A series of bursts was repeated three times after 5-min stabilization.

### Echocardiography

Two-dimensional M-mode echocardiography was performed on mice using a 30 MHz probe (Vevo 1100 system; VisualSonics, Toronto, ON, Canada) as described previously (Wang et al., 2018; Chen et al., 2019; Yan et al., 2019; Zhang et al., 2020). The left atrium (LA) chamber dimensions were measured.

### Histological Analysis

Atrial samples were fixed in 4% paraformaldehyde, embedded in paraffin, and then sectioned (5 μm). The staining of Hematoxylin and eosin (H&E) and Masson’s Trichrome were performed on the atrial sections in accordance with the standard procedure (Li et al., 2018, 2019; Zhang et al., 2020). The immunohistochemistry staining was performed with anti-α-smooth muscle actin (α-SMA) (1:200, Abcam, MA, United States) and anti-Mac-2 (1:200, Abcam) as described (Wang et al., 2018). Cryosections were stained with the dihydroethidine (DHE, 1 μM in PBS) for 30 min at 37°C. Fluorescence was detected using Nikon Labophot 2 microscope (Nikon, Tokyo, Japan).

### Proteasome Activity Assay

The activity of proteasome in the atria was gauged by specific luminogenic peptide substrates, including Z-nLPnLD-aminoluciferin, Z-LRR-aminoluciferin, and Suc-LLVY-aminoluciferin for the caspase-like, trypsin-like, and chymotrypsin-like activities according to the manufacturer’s instructions (Li et al., 2018, 2019; Chen et al., 2019).

### Quantitative Real-Time PCR Analysis

The quantitative real-time PCR (qPCR) was gauged by an iCycler IQ system (Bio-Rad, CA, United States). Total RNA from atrium tissues were isolated with TRIzol and reverse transcribed as described (Wang et al., 2016). The cDNA was used for PCR amplification with gene-specific primers (Sangon Biotech, Shanghai, China), including α-SMA, collagen I, collagen III, IL-1β, IL-6, TNF-α, MCP-1, NOX2, NOX4, β1, β2, β5, β1i, β2i, and β5i (Supplementary Table 1). The transcript quantities were normalized to the amount of endogenous control (GAPDH).

### Western Blot Analysis

Total proteins were extracted from snap-frozen atrium samples using RIPA buffer plus protease inhibitors (Solarbio Science Technology Co., China). The protein lysates were separated by electrophoresis in 8–12% SDS-PAGE gels, transferred to polyvinylidene difluoride (PVDF) membranes. The membranes were incubated with appropriate primary antibodies and then with horseradish peroxidase-conjugated secondary antibodies. The signal intensities of immunoblots were detected by the ECL Plus chemiluminescent system (Bio-Rad, CA, United States) and were analyzed with a Gel-pro 4.5 Analyzer (Media Cybernetics, United States) (Chen et al., 2019).

### Statistical Analysis

The normality test (Shapiro–Wilk) was utilized to verify whether the data were normally distributed. The student *t*-test was performed to evaluate the significant difference between two groups in normal distribution. For the data that were not normally distributed, the Mann–Whitney test was used. For the other comparisons, the significance of the difference between means of the groups was determined by one-way ANOVA following Newman–Keuls multiple comparison test from GraphPad Prism 5 (GraphPad Prism Software). The values of *P* < 0.05 were considered statistically significant.

## Results

### GA Inhibits Ang II-Induced AF in Mice

**FIGURE 1:**
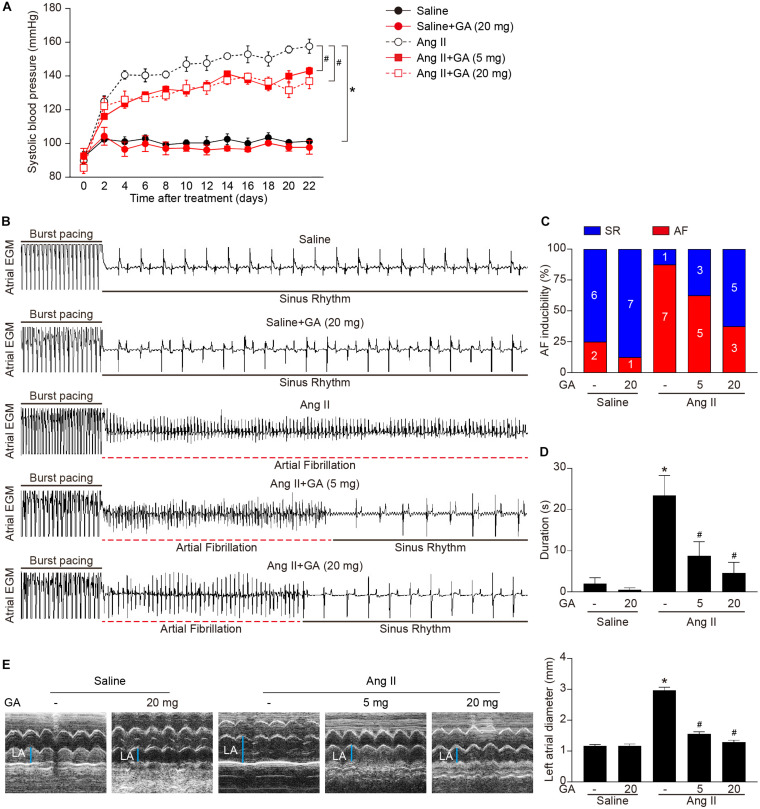
Administration of GA attenuates Ang II-induced AF and atrial dilation. Mice were infused with Ang II (2000 ng kg^– 1^ min^– 1^) for 21 days. **(A)** Systolic blood pressure (SBP) was measured by the non-invasive tail-cuff method in vehicle or GA-treated mice before and after Ang II infusion. **(B)** Representative atrial electrogram recordings. Burst pacing is highlighted by solid underlines, while dashed underlines indicate AF. **(C)** Percentage of mice in which AF was successfully achieved in each group (*n* = 8). **(D)** AF duration in mice with AF induction. **(E)** M-mode echocardiography of left atrium (LA) chamber (left), and quantification of LA diameter (right, *n* = 8). *P < 0.05 versus saline, ^#^*P* < 0.05 versus Ang II.

We first tested whether administration of GA could reduce AF. GA treatment significantly improved the elevation of SBP in Ang II-treated mice in a dose-dependent manner (Figure 1A). Moreover, the incidence and duration of Ang II-induced AF were significantly reduced in GA-treated mice compared with the vehicle-treated mice (Figures 1B–D). Furthermore, Ang II infusion resulted in a marked increase in LA dilation in mice, which was attenuated after GA treatment (Figure 1E). There was no significant difference in SBP, AF inducibility and duration, or LA dilation between the two groups after saline infusion (Figures 1A–E). Thus, these data suggest that GA administration attenuates Ang II-induced AF.

### GA Suppresses Ang II-Induced Atrial Fibrosis

**FIGURE 2:**
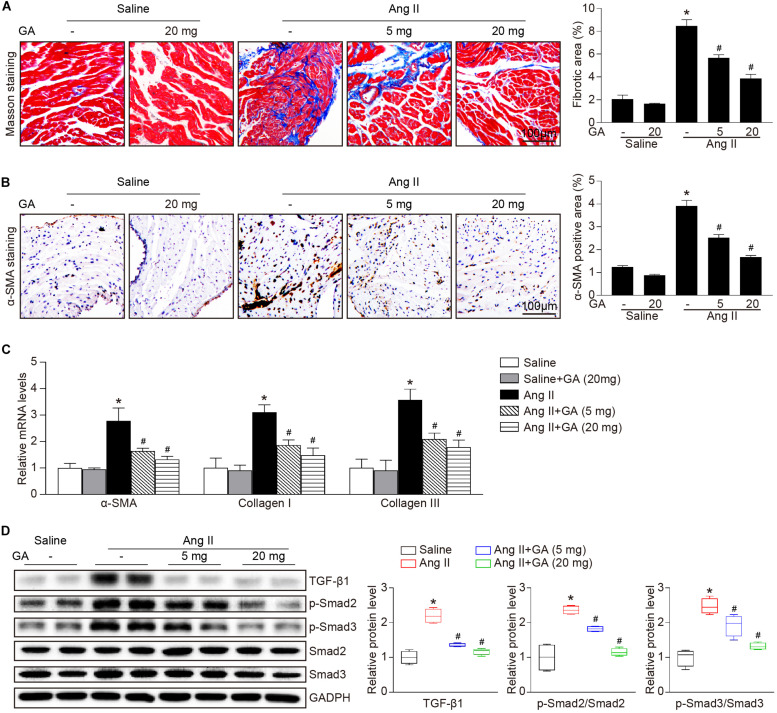
GA inhibits Ang II-induced atrial remodeling. **(A)** Representative images of Masson trichrome staining for atrial fibrosis (left). Quantification of fibrotic area (right, *n* = 6). Scale bar: 100 μm. **(B)** Representative images of immunohistochemical staining of LA sections to detect myofibroblast activation (left). Quantification of α-SMA^+^ areas (right, *n* = 6). Scale bar: 100 μm. **(C)** qPCR analyses of the mRNA expression levels of α-SMA, collagen I, and collagen III (*n* = 6). **(D)** Immunoblotting analysis of the protein levels of TGF-β1, p-Smad2/3, and Smad2/3 in the atria (left), and quantification of each protein band (right, *n* = 4). GAPDH as an internal control. *P < 0.05 versus saline, ^#^*P* < 0.05 versus Ang II.

We next examined whether GA attenuates the formation of atrial fibrosis. After 3 weeks of Ang II infusion, the atrial fibrotic area, percentage of α-SMA^+^ myofibroblasts, and mRNA expression of α-SMA, collagen I, and collagen III were increased in mice, and these effects were significantly abrogated by GA administration (Figures 2A–C). Moreover, Ang II infusion markedly upregulated the key signaling mediators of atrial fibrosis, including TGF-β1 and p-Smad2/3, while this effect was diminished in GA-treated mice (Figure 2D). Accordingly, these results indicate that GA treatment effectively improves adverse atrial structural remodeling after Ang II infusion.

### GA Attenuates Ang II-Induced Atrial Inflammation and Oxidative Stress

**FIGURE 3:**
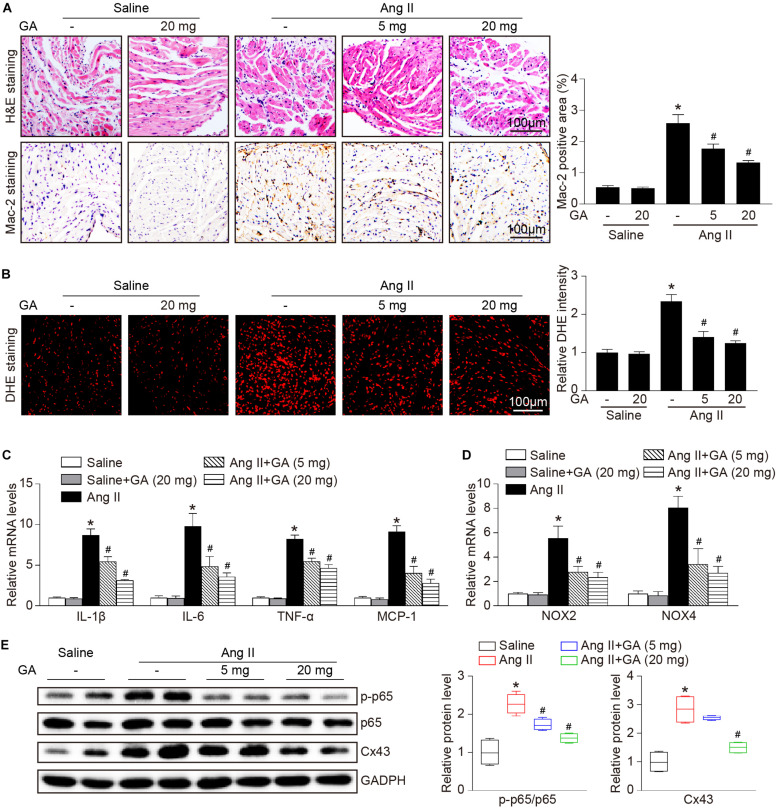
GA reduces atrial inflammation and oxidative stress in mice after Ang II infusion. **(A)** Representative H&E staining (top) and Mac-2 immunohistochemistry (bottom) in the atria. Quantification of Mac-2–positive cells (right, *n* = 6). Scale bar: 100 μm. **(B)** DHE staining of atrial superoxide production (left), and quantification of DHE intensity (right, *n* = 6). Scale bar: 100 μm. **(C)** qPCR analyses of the mRNA expression levels of IL-1β, IL-6, TNF-α, and MCP-1 (*n* = 6). **(D)** qPCR analyses of the mRNA expression of NOX2 and NOX4. **(E)** Immunoblotting analysis of the protein expression levels of p-p65, p65, and Cx43 in the atria (left), and quantification of each protein band (right, *n* = 4). GAPDH as an internal control. *P < 0.05 versus saline, ^#^*P* < 0.05 versus Ang II.

The administration of GA dose-dependently inhibited Ang II-induced infiltration of inflammatory cells (as indicated by Mac-2-positive macrophages) and the mRNA expression levels of IL-1β, IL-6, TNF-α, and MCP-1 compared with the vehicle (Figures 3A,C). Furthermore, Ang II infusion increased atrial superoxide formation (demonstrated by DHE staining) and the gene expression of NOX2 and NOX4 (Figures 3B,D). These effects were reduced in GA-treated mice (Figures 3B,D). We next determined the effect of GA on NF-κB signaling and gap junction gene expression. In agreement with our recent studies (Li et al., 2018, 2019; Zhang et al., 2020), the protein expression levels of p-p65 and connexin 43 (Cx43) were upregulated in Ang II-treated atria, and this upregulation was reversed after GA treatment (Figure 3E).

### GA Inhibits Immunoproteasome Activity and PTEN Degradation

**FIGURE 4:**
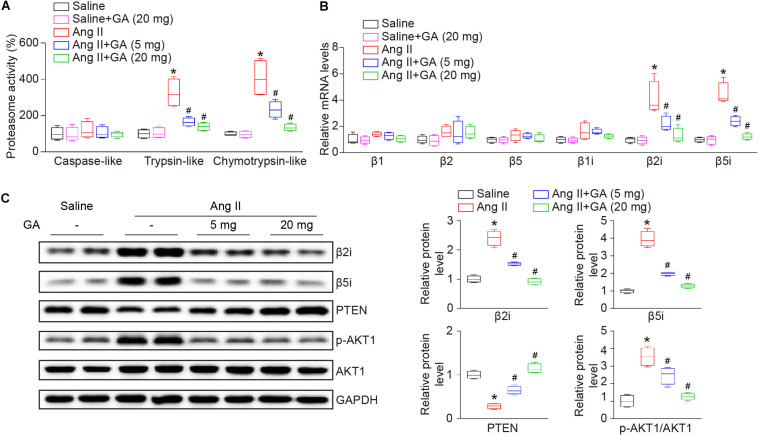
GA reduces the activity and expression of immunoproteasome subunits β2i and β5i and PTEN degradation in the atria after Ang II infusion. **(A)** The proteasome activities in the atria after Ang II infusion in the presence or absence of GA treatment (*n* = 4). **(B)** qPCR analyses of the mRNA expression levels of β1, β2, β5, β1i, β2i, and β5i (*n* = 4). **(C)** Representative immunoblotting analyses of the protein expression levels of β2i, β5i, PTEN, p-AKT1, and AKT1 (left), and quantification of the relative protein levels (right, *n* = 4). GAPDH as an internal control. *P < 0.05 versus saline, ^#^*P* < 0.05 versus Ang II.

Recent studies have demonstrated that PTEN and its downstream signaling are involved in Ang II-induced AF (Li et al., 2018, 2019), therefore next we investigated the effect of GA on proteasome-mediated PTEN degradation in the atria. The administration of GA significantly and dose-dependently suppressed Ang II-induced trypsin-like and chymotrypsin-like activities (Figure 4A). Because constitutively expressed β-subunits (β1, β2, and β5) and immunosubunits (β1i, β2i, and β5i) have proteasome activity, RT-PCR and western blotting analysis were utilized to evaluate subunit expression. Ang II infusion increased the mRNA expression levels of β2i and β5i compared with saline control (Figure 4B). Furthermore, GA treatment dose-dependently reduced the gene expression of the immunosubunits β2i and β5i but not β1i and constitutively expressed β-subunits (β1, β2, and β5) in the atria after Ang II infusion (Figure 4B). In addition, the Ang II-induced increase in protein expression levels of β2i and β5i was also downregulated in GA-treated atria tissues (Figure 4C). In line with our previous findings (Li et al., 2018, 2019), Ang II infusion for 3 weeks increased PTEN degradation and p-AKT1 protein expression, and these effects were dose-dependently reversed by the administration of GA (Figure 4C). Taken together, these results demonstrate that GA treatment reduces PTEN degradation likely via suppressing the expression and activity of immunoproteasome subunits in Ang II-treated atria.

### Blockage of PTEN Activity Reduces the GA-Mediated Protective Effect on Ang II-Induced AF

**FIGURE 5:**
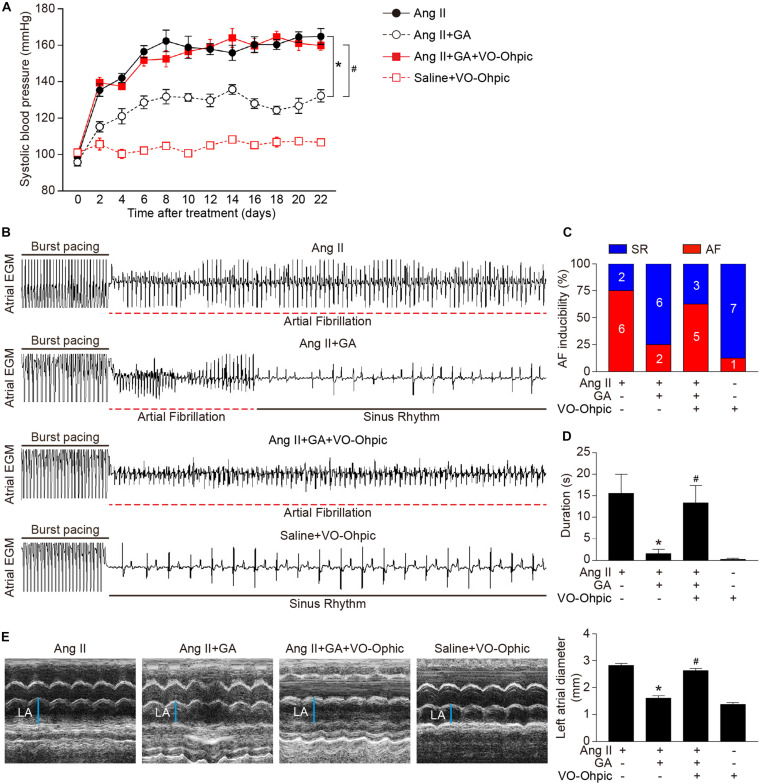
Blocking of PTEN activity suppresses the GA-mediated beneficial effect on Ang II-induced AF. **(A)** SBP was measured by the non-invasive tail-cuff method in the vehicle or GA-treated mice before and after Ang II infusion with or without VO-OHpic treatment. **(B)** Representative atrial electrogram recordings. Burst pacing is highlighted by solid underlines, while dashed underlines indicate AF. **(C)** Percentage of mice in which AF was successfully achieved in each group (*n* = 8). **(D)** AF duration in mice with AF induction. **(E)** M-mode echocardiography of LA chamber (left), and quantification of LA diameter (right, *n* = 8). *P < 0.05 versus Ang II, ^#^*P* < 0.05 versus Ang II + GA.

**FIGURE 6:**
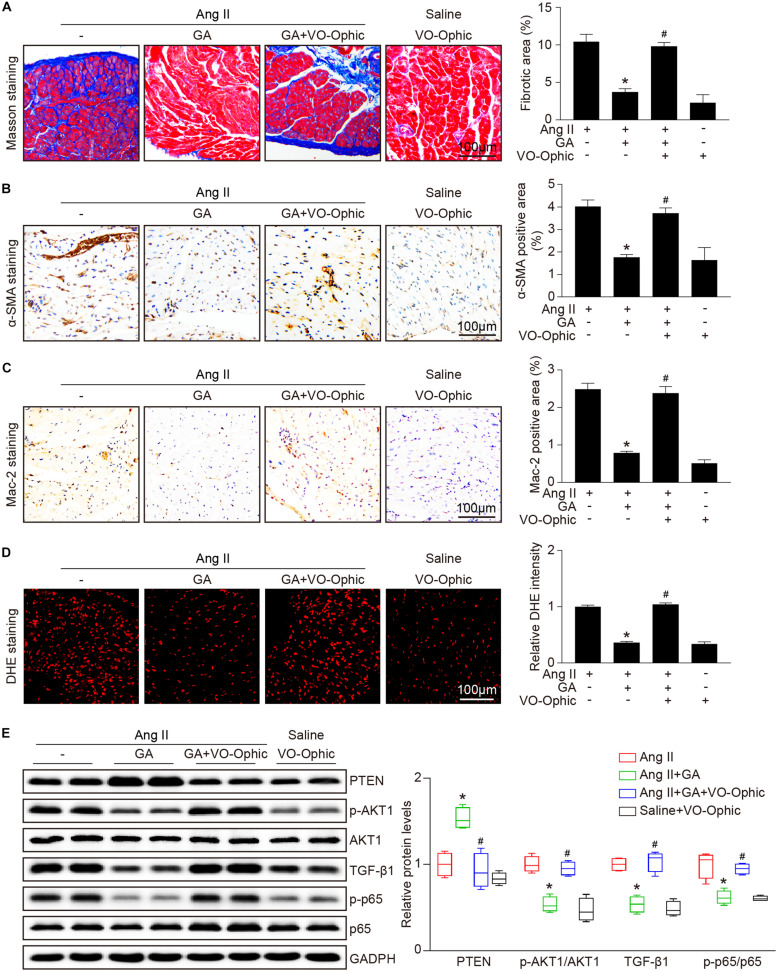
PTEN inhibition blocks the GA-mediated protective effect on atrial remodeling and downstream signaling pathways. **(A)** Representative images of Masson trichrome staining for atrial fibrosis (left). Quantification of fibrotic area (right, *n* = 6). Scale bar: 100 μm. **(B)** Representative images of immunohistochemical staining of LA sections to detect myofibroblast activation (left). Quantification of α-SMA^+^ areas (right, *n* = 6). Scale bar: 100 μm. **(C)** Representative Mac-2 (immunohistochemistry (left) in the atria. Scale bar: 100 μm. Quantification of Mac-2–positive cells (right, *n* = 6). **(D)** DHE staining of atrial superoxide production (left), and quantification of DHE intensity (right, *n* = 6). Scale bar: 100 μm. **(E)** Representative immunoblotting analyses of the protein expression levels of PTEN, p-AKT1, AKT1, TGF-β1, p-p65, and p65 (left), and quantification of the relative protein levels (right, *n* = 4). GAPDH as an internal control. *P < 0.05 versus Ang II, ^#^*P* < 0.05 versus Ang II + GA.)

To identify the role of PTEN in Ang II-induced atrial remodeling and AF after GA treatment, mice were treated with the PTEN specific inhibitor VO-OHpic with or without administration of GA. Three weeks after Ang II infusion, mice treated with GA exhibited a significant reduction of SBP, AF inducibility and duration, and LA dilation compared with the vehicle-treated mice (Figures 5A–E); these effects were abolished by VO-OHpic treatment (Figures 5A–E). Furthermore, the GA-mediated decrease in atrial fibrotic area, number of α-SMA-positive myofibroblasts, Mac-2^+^ macrophage infiltration, superoxide production, and mRNA expression levels of α-SMA, collagen I, and collagen III were reversed by VO-OHpic in GA-treated mice (Figures 6A–D and Supplementary Figure 1). Accordingly, GA-induced reduction of PTEN degradation and protein expression of p-AKT1, TGF-β1, and p-p65 in Ang II-treated mice were blocked after VO-OHpic treatment (Figure 6E). Collectively, these results suggest that GA inhibits Ang II-induced atrial remodeling and AF via reducing PTEN degradation.

## Discussion

**FIGURE 7:**
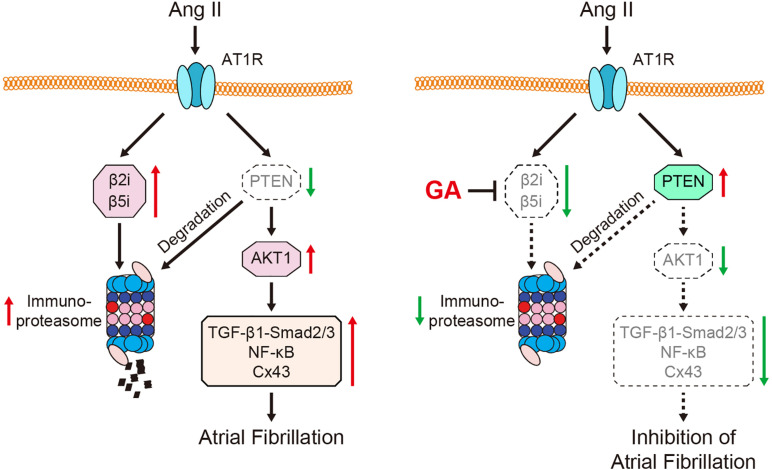
A schematic model of the GA-mediated cardioprotection in Ang II-induced AF. GA inactivates immunoproteasome-dependent degradation of PTEN, leading to the downregulation of AKT1, TGF-β1-Smad2/3, NF-κB p65, and Cx43 signaling mediators. Therefore, GA ameliorates atrial remodeling and AF induced by Ang II infusion.

Here, we revealed for the first time that the administration of GA inhibits Ang II-induced AF incidence and atrial dilation. Specifically, GA blocks the activity and expression of the immunoproteasome catalytic subunit β2i and β5i, which reduces PTEN degradation and AKT activation. This leads to the suppression of downstream signaling mediators (TGF-β1-Smad2/3, NF-κB, and Cx43), which improves atrial fibrosis, inflammation, and oxidative stress (Figure 7). Therefore, our results provide new evidence that GA serves as an effective inhibitor of the immunoproteasome and is a promising agent for treating AF and atrial structural remodeling.

Atrial structural remodeling is the hallmark of the development and progression of AF, which subsequently causes LA enlargement and conduction abnormalities (Andrade et al., 2014). This process consists of several mechanisms, including atrial fibrosis, inflammation, and oxidative stress (Dzeshka et al., 2015). Ang II has been demonstrated to significantly increase SBP, left ventricular hypertrophy, and dysfunction, which are established clinical risk factors for AF (Purohit et al., 2013; Jansen et al., 2018). Current therapeutic agents for AF, which include oral anticoagulation, angiotensin-converting enzyme inhibitors, and antiarrhythmic drugs, focus on the common symptoms and complications. However, adverse events with these agents may increase mortality (Kirchhof, 2017). Therefore, new options targeting atrial structural remodeling, atrial dilation, and AF are urgently needed. GA is a natural polyphenol compound that exerts a key role in protecting against cardiac hypertrophy, hypertension, and fibrosis in several animal models (Jin et al., 2017, 2018b). Recently, we demonstrated that GA administration improved pressure overload-induced myocardial hypertrophic remodeling (including hypertrophy, fibrosis, inflammation, and oxidative stress) (Yan et al., 2019) and Ang II-induced hypertension and vascular dysfunction (Yan et al., 2020), suggesting that GA may exert a protective effect on AF. In agreement with this, we demonstrated that GA not only markedly reduced the elevation blood pressure but also attenuated atrial fibrosis, inflammation, and ROS production in Ang II-treated mice (Figures 2, 3), which are involved in the pathogenesis of AF. Thus, our results indicate that GA markedly reduced AF development at least in part through blocking Ang II/hypertension-induced atrial remodeling.

Proteasomal degradation is the vital pathway for intracellular protein turnover in most mammalian cells and organs, including the heart (Lyon et al., 2013). After stimulation by inflammatory cytokines, H_2_O_2_, and heat shock, the immunoproteasome is induced and exhibits immune and non-immune functions, such as antigen presentation, inflammation, oxidative stress, and cell signaling (Angeles et al., 2012). Increasing evidence demonstrates that adverse stimuli including pressure overload, isoproterenol, deoxycortone acetate/salt, and Ang II increase the catalytic activity and expression of the immunoproteasome, leading to cardiac hypertrophy and AF (Depre et al., 2006; Drews et al., 2010; Li et al., 2015; Yan et al., 2017). In contrast, the proteasome inhibitor bortezomib effectively ameliorates Ang II-induced myocardial hypertrophy and contractile function (Li et al., 2015). Moreover, we recently found that the activity and expression of β2i and β5i are upregulated in the atria of Ang II-treated mice and the serum of patients with AF (Li et al., 2018, 2019). Knockout of β2i or β5i attenuates Ang II-induced atrial remodeling and development of AF (Li et al., 2018, 2019). Interestingly, several natural products, including quercetin and resveratrol, have been shown to inhibit proteasome activity, thereby ameliorating atherosclerosis and cardiac hypertrophy (Pashevin et al., 2011; Chen et al., 2019). In the present study, we have provided novel evidence that GA administration significantly suppresses the activity and expression of the immunoproteasome catalytic subunits β2i and β5i in the atria of Ang II-treated mice.

Accumulating evidence suggests that PTEN plays a critical role in cardiovascular disease by inhibiting AKT-dependent pathways (GSK3, FOXO, and mTOR) and PINK1-AMPK signaling cascades (Crackower et al., 2002; Song et al., 2012; Xu et al., 2014; Roe et al., 2015). The cardiomyocyte-specific dysfunction of PTEN in mice results in myocardial hypertrophy and contractility defects, which are accompanied by inhibition of autophagy (Crackower et al., 2002; Xu et al., 2014; Roe et al., 2015). However, these effects could be reversed by rapamycin-induced suppression of mTOR and metformin-dependent activation of AMPK (Xu et al., 2014; Roe et al., 2015). Of note, our recent studies have demonstrated that PTEN plays a role in Ang II-induced AF and atrial structural remodeling by ameliorating AKT1 and downstream signaling mediators (TGF-β1-Smad2/3, NF-κB, and Cx43) (Li et al., 2018, 2019). Thus, the regulation of PTEN activity and/or protein expression is considered as a promising therapeutic strategy for heart disease. PTEN activity is indeed modulated by post-translational modifications, especially ubiquitination-mediated proteasomal degradation (Lee et al., 2018). We and others demonstrated that proteasomal dysfunction induced by knockout of the immunoproteasome subunits β1i, β2i, or β5i in mice attenuated PTEN degradation, leading to the downregulation of AKT-associated signaling pathways in ischemic hearts and AF (Cai et al., 2008; Li et al., 2018, 2019). Interestingly, several natural compounds have been identified as potent inhibitors of PTEN degradation. Indole-3-carbinol, derived from cruciferous vegetables, directly blocks E3 ubiquitin ligase WWP1 and subsequently suppresses the K27-linked ubiquitination of PTEN (Lee et al., 2019). Moreover, we recently showed that resveratrol represents a novel inhibitor of the immunoproteasome and restores PTEN stability in pressure overload-induced cardiac hypertrophy and fibrosis (Chen et al., 2019; Zou et al., 2019). In the present study, we extended our previous findings and demonstrated that administration of GA markedly decreased PTEN degradation and activation of AKT1, leading to inhibition of AF and atrial remodeling in Ang II-treated mice. These effects were reversed by the specific PTEN inhibitor VO-OHpic. Collectively, our novel findings suggest that PTEN plays a role in the GA-mediated cardioprotective effects on Ang II-treated atria.

Several studies have explored the bioavailability of GA in humans (Manach et al., 2005). After the oral administration of pure tablets or black tea (each containing 50 mg GA), GA was rapidly absorbed and the plasma concentrations of GA reached 1.83 μmol/L (Shahrzad et al., 2001). Interestingly, our recent study revealed that the dosage of GA used in mice (5 or 20 mg/kg BW) was equivalent to 0.41 or 1.63 mg kg/BW in humans (Yan et al., 2019), which is consistent with the results of others. Moreover, we found that primary cardiomyocytes treated with GA (1–200 μmol) showed no significant toxic effect (Yan et al., 2019). Thus, GA may serve as a safe and effective natural product for the prevention and treatment of cardiovascular diseases. Further investigations are needed to verify the bioavailability of different forms of GA in foods.

## Conclusion

In summary, in this study we demonstrated for the first time that GA administration significantly attenuated Ang II-induced AF and atrial structural remodeling in mice. Moreover, GA suppressed the activity and expression of the immunoproteasome subunits β2i and β5i, which ameliorated PTEN degradation leading to inactivation of AKT1 and downstream signaling pathways (TGF-β1-Smad2/3, NF-κB, and Cx43). Thus, these findings identify GA as a novel inhibitor of the immunoproteasome and suggest a potential approach to AF prevention and treatment.

## Data Availability Statement

The data that support the findings of this study are available from the corresponding author upon reasonable request.

## Ethics Statement

The animal study was reviewed and approved by the Animal Care and Use Committee of Dalian Medical University (No. LCKY2016-31) and conformed to the US National Institutes of Health Guide for the Care and Use of Laboratory Animals.

## Author Contributions

DH, Q-YZ, Y-LZ, and XH conducted the experiments. DH, FT, and XY analyzed the data. S-BG, XY, and H-HL designed the study. XY and H-HL drafted the manuscript, provided the funding for the study, and had primary responsibility for the final content. All authors contributed to the article and approved the submitted version.

## Conflict of Interest

The authors declare that the research was conducted in the absence of any commercial or financial relationships that could be construed as a potential conflict of interest.

## References

[B1] Andrade J. Khairy P. Dobrev D. Nattel S. (2014). The clinical profile and pathophysiology of atrial fibrillation: relationships among clinical features, epidemiology, and mechanisms. *Circ. Res.* 114 1453–1468. 10.1161/CIRCRESAHA.114.303211 24763464

[B2] Angeles A. Fung G. Luo H. (2012). Immune and non-immune functions of the immunoproteasome. *Front. Biosci. (Landmark Ed)* 17:4027. 10.2741/4027 22201844

[B3] Cai Z. P. Shen Z. Van Kaer L. Becker L. C. (2008). Ischemic preconditioning-induced cardioprotection is lost in mice with immunoproteasome subunit low molecular mass polypeptide-2 deficiency. *FASEB J.* 22 4248–4257. 10.1096/fj.08-105940 18728217 PMC2614607

[B4] Chen C. Zou L. X. Lin Q. Y. Yan X. Bi H. L. Xie X. (2019). Resveratrol as a new inhibitor of immunoproteasome prevents PTEN degradation and attenuates cardiac hypertrophy after pressure overload. *Redox Biol.* 20 390–401. 10.1016/j.redox.2018.10.021 30412827 PMC6226597

[B5] Crackower M. A. Oudit G. Y. Kozieradzki I. Sarao R. Sun H. Sasaki T. (2002). Regulation of myocardial contractility and cell size by distinct PI3K-PTEN signaling pathways. *Cell* 110 737–749. 10.1016/s0092-8674(02)00969-8 12297047

[B6] Depre C. Wang Q. Yan L. Hedhli N. Peter P. Chen L. (2006). Activation of the cardiac proteasome during pressure overload promotes ventricular hypertrophy. *Circulation* 114 1821–1828. 10.1161/CIRCULATIONAHA.106.637827 17043166

[B7] Drews O. Tsukamoto O. Liem D. Streicher J. Wang Y. Ping P. (2010). Differential regulation of proteasome function in isoproterenol-induced cardiac hypertrophy. *Circ. Res.* 107 1094–1101. 10.1161/CIRCRESAHA.110.222364 20814020 PMC3360925

[B8] Dzeshka M. S. Lip G. Y. Snezhitskiy V. Shantsila E. (2015). Cardiac fibrosis in patients with atrial fibrillation: mechanisms and clinical mmplications. *J. Am. Coll. Cardiol.* 66 943–959. 10.1016/j.jacc.2015.06.1313 26293766

[B9] Gao G. Dudley S. C. Jr. (2009). Redox regulation, NF-kappaB, and atrial fibrillation. *Antioxid. Redox Signal.* 11 2265–2277. 10.1089/ARS.2009.2595 19309257 PMC2819799

[B10] Hu Y. F. Chen Y. J. Lin Y. J. Chen S. A. (2015). Inflammation and the pathogenesis of atrial fibrillation. *Nat. Rev. Cardiol.* 12 230–243. 10.1038/nrcardio.2015.2 25622848

[B11] Jansen H. J. Mackasey M. Moghtadaei M. Belke D. D. Egom E. E. Tuomi J. M. (2018). Distinct patterns of atrial electrical and structural remodeling in angiotensin II mediated atrial fibrillation. *J. Mol. Cell. Cardiol.* 124 12–25. 10.1016/j.yjmcc.2018.09.011 30273558

[B12] Jin L. Piao Z. H. Liu C. P. Sun S. Liu B. Kim G. R. (2018a). Gallic acid attenuates calcium calmodulin-dependent kinase II-induced apoptosis in spontaneously hypertensive rats. *J. Cell. Mol. Med.* 22 1517–1526. 10.1111/jcmm.13419 29266709 PMC5824377

[B13] Jin L. Piao Z. H. Sun S. Liu B. Kim G. R. Seok Y. M. (2017). Gallic acid reduces blood pressure and attenuates oxidative stress and cardiac hypertrophy in spontaneously hypertensive rats. *Sci. Rep.* 7:15607. 10.1038/s41598-017-15925-1 29142252 PMC5688141

[B14] Jin L. Sun S. Ryu Y. Piao Z. H. Liu B. Choi S. Y. (2018b). Gallic acid improves cardiac dysfunction and fibrosis in pressure overload-induced heart failure. *Sci. Rep.* 8:9302. 10.1038/s41598-018-27599-4 29915390 PMC6006337

[B15] Kirchhof P. (2017). The future of atrial fibrillation management: integrated care and stratified therapy. *Lancet* 390 1873–1887. 10.1016/S0140-6736(17)31072-3 28460828

[B16] Lee Y. R. Chen M. Lee J. D. Zhang J. Lin S. Y. Fu T. M. (2019). Reactivation of PTEN tumor suppressor for cancer treatment through inhibition of a MYC-WWP1 inhibitory pathway. *Science* 364:eaau0159. 10.1126/science.aau0159 31097636 PMC7081834

[B17] Lee Y. R. Chen M. Pandolfi P. P. (2018). The functions and regulation of the PTEN tumour suppressor: new modes and prospects. *Nat. Rev. Mol. Cell Biol.* 19 547–562. 10.1038/s41580-018-0015-0 29858604

[B18] Li J. Wang S. Bai J. Yang X. L. Zhang Y. L. Che Y. L. (2018). Novel role for the immunoproteasome subunit PSMB10 in angiotensin II-induced atrial fibrillation in mice. *Hypertension* 71 866–876. 10.1161/HYPERTENSIONAHA.117.10390 29507100

[B19] Li J. Wang S. Zhang Y. L. Bai J. Lin Q. Y. Liu R. S. (2019). Immunoproteasome subunit beta5i promotes Ang II (Angiotensin II)-induced atrial fibrillation by targeting ATRAP (Ang II type I receptor-associated protein) degradation in mice. *Hypertension* 73 92–101. 10.1161/HYPERTENSIONAHA.118.11813 30571551

[B20] Li N. Wang H. X. Han Q. Y. Li W. J. Zhang Y. L. Du J. (2015). Activation of the cardiac proteasome promotes angiotension II-induced hypertrophy by down-regulation of ATRAP. *J. Mol. Cell. Cardiol.* 79 303–314. 10.1016/j.yjmcc.2014.12.007 25526681

[B21] Lyon R. C. Lange S. Sheikh F. (2013). Breaking down protein degradation mechanisms in cardiac muscle. *Trends Mol. Med.* 19 239–249. 10.1016/j.molmed.2013.01.005 23453282 PMC3622835

[B22] Manach C. Williamson G. Morand C. Scalbert A. Remesy C. (2005). Bioavailability and bioefficacy of polyphenols in humans. I. Review of 97 bioavailability studies. *Am. J. Clin. Nutr.* 81(Suppl. 1) 230S–242S. 10.1093/ajcn/81.1.230S 15640486

[B23] Pashevin D. A. Tumanovska L. V. Dosenko V. E. Nagibin V. S. Gurianova V. L. Moibenko A. A. (2011). Antiatherogenic effect of quercetin is mediated by proteasome inhibition in the aorta and circulating leukocytes. *Pharmacol. Rep.* 63 1009–1018. 10.1016/s1734-1140(11)70617-x 22001989

[B24] Purohit A. Rokita A. G. Guan X. Chen B. Koval O. M. Voigt N. (2013). Oxidized Ca(2+)/calmodulin-dependent protein kinase II triggers atrial fibrillation. *Circulation* 128 1748–1757. 10.1161/CIRCULATIONAHA.113.003313 24030498 PMC3876034

[B25] Roe N. D. Xu X. Kandadi M. R. Hu N. Pang J. Weiser-Evans M. C. (2015). Targeted deletion of PTEN in cardiomyocytes renders cardiac contractile dysfunction through interruption of Pink1-AMPK signaling and autophagy. *Biochim. Biophys. Acta* 1852 290–298. 10.1016/j.bbadis.2014.09.002 25229693 PMC4277923

[B26] Ryu Y. Jin L. Kee H. J. Piao Z. H. Cho J. Y. Kim G. R. (2016). Gallic acid prevents isoproterenol-induced cardiac hypertrophy and fibrosis through regulation of JNK2 signaling and Smad3 binding activity. *Sci. Rep.* 6:34790. 10.1038/srep34790 27703224 PMC5050511

[B27] Shahrzad S. Aoyagi K. Winter A. Koyama A. Bitsch I. (2001). Pharmacokinetics of gallic acid and its relative bioavailability from tea in healthy humans. *J. Nutr.* 131 1207–1210. 10.1093/jn/131.4.1207 11285327

[B28] Song M. S. Salmena L. Pandolfi P. P. (2012). The functions and regulation of the PTEN tumour suppressor. *Nat. Rev. Mol. Cell Biol.* 13 283–296. 10.1038/nrm3330 22473468

[B29] Staerk L. Sherer J. A. Ko D. Benjamin E. J. Helm R. H. (2017). Atrial fibrillation: epidemiology. pathophysiology, and clinical outcomes. *Circ. Res.* 120 1501–1517. 10.1161/CIRCRESAHA.117.309732 28450367 PMC5500874

[B30] Wang L. Zhang Y. L. Lin Q. Y. Liu Y. Guan X. M. Ma X. L. (2018). CXCL1-CXCR2 axis mediates angiotensin II-induced cardiac hypertrophy and remodelling through regulation of monocyte infiltration. *Eur. Heart J.* 39 1818–1831. 10.1093/eurheartj/ehy085 29514257

[B31] Wang L. Zhao X. C. Cui W. Ma Y. Q. Ren H. L. Zhou X. (2016). Genetic and pharmacologic inhibition of the chemokine receptor CXCR2 prevents experimental hypertension and vascular dysfunction. *Circulation* 134 1353–1368. 10.1161/CIRCULATIONAHA.115.020754 27678262 PMC5084654

[B32] Xu X. Roe N. D. Weiser-Evans M. C. Ren J. (2014). Inhibition of mammalian target of rapamycin with rapamycin reverses hypertrophic cardiomyopathy in mice with cardiomyocyte-specific knockout of PTEN. *Hypertension* 63 729–739. 10.1161/HYPERTENSIONAHA.113.02526 24446058

[B33] Yan W. Bi H. L. Liu L. X. Li N. N. Liu Y. Du J. (2017). Knockout of immunoproteasome subunit beta2i ameliorates cardiac fibrosis and inflammation in DOCA/Salt hypertensive mice. *Biochem. Biophys. Res. Commun.* 490 84–90. 10.1016/j.bbrc.2017.05.011 28478040

[B34] Yan X. Zhang Q.-Y. Zhang Y.-L. Han X. Guo S.-B. Li H.-H. (2020). Gallic acid attenuates angiotensin II-induced hypertension and vascular dysfunction by inhibiting the degradation of endothelial nitric oxide synthase. *Front. Pharmacol.* 11:1121. 10.3389/fphar.2020.01121 32848742 PMC7396711

[B35] Yan X. Zhang Y. L. Zhang L. Zou L. X. Chen C. Liu Y. (2019). Gallic acid suppresses cardiac hypertrophic remodeling and heart failure. *Mol. Nutr. Food. Res.* 63:e1800807. 10.1002/mnfr.201800807 30521107

[B36] Zhang Y. L. Cao H. J. Han X. Teng F. Chen C. Yang J. (2020). Chemokine receptor CXCR-2 initiates atrial fibrillation by triggering monocyte mobilization in mice. *Hypertension* 76 381–392. 10.1161/HYPERTENSIONAHA.120.14698 32639881

[B37] Zou L. X. Chen C. Yan X. Lin Q. Y. Fang J. Li P. B. (2019). Resveratrol attenuates pressure overload-induced cardiac fibrosis and diastolic dysfunction via PTEN/AKT/Smad2/3 and NF-kappaB signaling pathways. *Mol. Nutr. Food Res.* 63:e1900418. 10.1002/mnfr.201900418 31655498

