# Supplementary figures and images for: Gallic Acid Ameliorates Angiotensin II-Induced Atrial Fibrillation by Inhibiting Immunoproteasome- Mediated PTEN Degradation in Mice
Source: Front Cell Dev Biol. 2020 Oct 30;8:594683. doi: 10.3389/fcell.2020.594683 (PMC7673442; doi:10.3389/fcell.2020.594683)

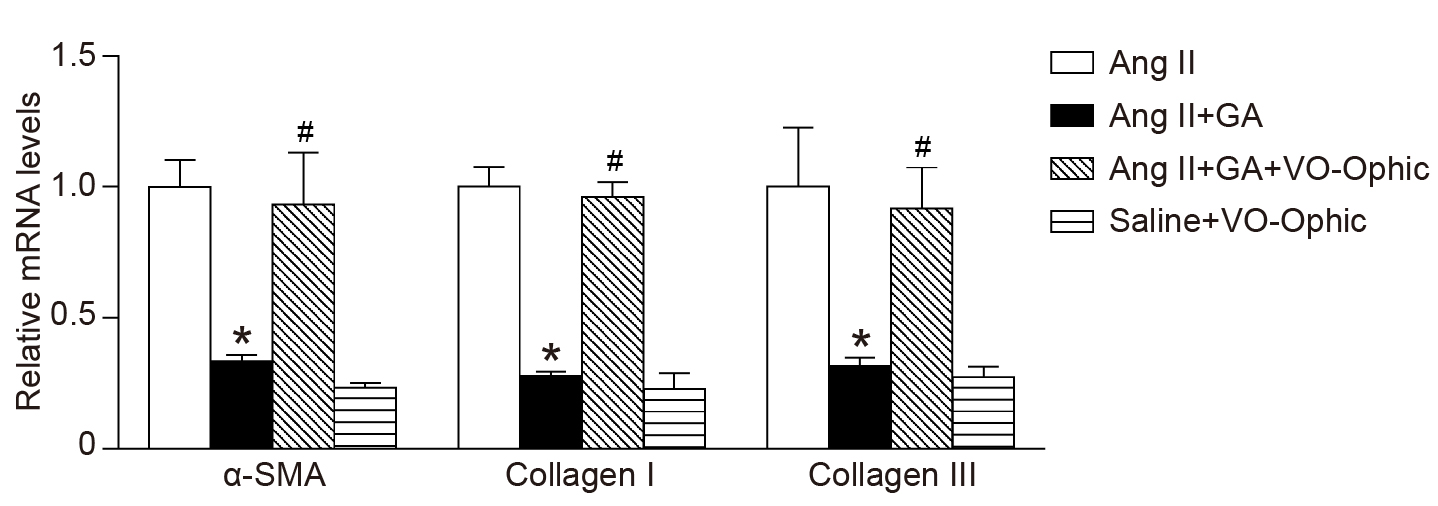

Supplement: Supplementary file 1 [file Image_1.JPEG]
