# Supplementary material for: Gallic Acid Ameliorates Angiotensin II-Induced Atrial Fibrillation by Inhibiting Immunoproteasome- Mediated PTEN Degradation in Mice
Source: Front Cell Dev Biol. 2020 Oct 30;8:594683. doi: 10.3389/fcell.2020.594683 (PMC7673442; doi:10.3389/fcell.2020.594683)
Supplement: Supplementary file 2 [file Table_1.DOCX]

**Supplementary Table 1**. Primer sequences for RT-PCR analysis

α-SMA, α-smooth muscle actin; IL-1β, interleukin-1β; IL-6, interleukin-6; TNF-α, tumor necrosis factor-α; MCP-1, monocyte chemoattractant protein-1; NOX2, NADPH oxidase 2; NOX4, NADPH oxidase 4; GAPDH, glyceraldehyde 3-phosphate dehydrogenase.

| Gene | Forward primer (5’-3’) | Reverse primer (5’-3’) |
| --- | --- | --- |
| α-SMA | TCCTGACGCTGAAGTATCCGATA | GGCCACACGAAGCTCGTTAT |
| Collagen I | GAGTACTGGATCGACCCTAACCA | GACGGCTGAGTAGGGAACACA |
| Collagen III | TCCCCTGGAATCTGTGAATC | TGAGTCGAATTGGGGAGAAT |
| IL-1β | CTTCCCCAGGGCATGTTAAG | ACCCTGAGCGACCTGTCTTG |
| IL-6 | TTCCATCCAGTTGCCTTCTTG | TTGGGAGTGGTATCCTCTGTGA |
| TNF-α | ATGGCCTCCCTCTCATCAGT | CTTGGTGGTTTGCTACGACG |
| MCP-1 | AGCTGTAGTTTTTGTCACCAAGC | GTGCTGAAGACCTTAGGGCA |
| NOX2 | CTTCTTGGGTCAGCACTGGC | GCAGCAAGATCAGCATGCAG |
| NOX4 | CTTGGTGAATGCCCTCAACT | TTCTGGGATCCTCATTCTGG |
| β1 | CCAATCGAGTGACTGACAAGCT | GGACTAGTGGAGGCTCGTTCA |
| β2 | AGGCCAGATATGGAGGAGGAA | GGGCACTGAGAATGGACGAA |
| β5 | TGCTCGCTAACATGGTGTATCAGTA | AGCCAGAGCCCACTGAGAAG |
| β1i | CTGGAGCTACACGGGTTGGA | ATATACCTGTCCCCCCTCACATT |
| β2i | CAGCCGTCTGCCCTTTACTG | AGAGCCCAGGTCACTCAGGAT |
| β5i | CTTGGCACCATGTCTGGTTGT | CCGGTACTGCAGCATCATGT |
| GAPDH | GGTTGTCTCCTGCGACTTCA | GGTGGTCCAGGGTTTCTTACTC |
